# Nano-Sized PtRu/C Electrocatalyst With Separated Phases and High Dispersion Improves Electrochemical Performance of Hydrogen Oxidation Reaction

**DOI:** 10.3389/fchem.2022.885965

**Published:** 2022-05-31

**Authors:** Yiling Feng, Wei Han, Tingyu Wang, Qian Chen, Yan Zhang, Yonggang Sun, Xin Zhang, Lin Yang, Song Chen, YuXiang Xu, Hong Tang, Bing Zhang, Hao Wang

**Affiliations:** ^1^ School of Chemistry and Chemical Engineering, Yancheng Institute of Technology, Yancheng, China; ^2^ School of Chemistry and Chemical Engineering, Guizhou Minzu University, Guizhou, China; ^3^ Jiangsu Ancan Technology Co., Ltd, Yancheng, China

**Keywords:** PtRu/C, nanoparticles, separated phases, hydrogen oxidation reaction, NaOH

## Abstract

Alloys and core-shell nanoparticles have recently received enormous attention which opened up new avenues for highly active catalysts. Despite considerable advances in this field, the majority of proposed approaches suffer from either complicated procedures or unstable structures, severely hindering their practical applications. Here, we successfully synthesized alloy electrocatalyst with separated phases, PtRu alloy nanoparticles robustly supported by carbon matrix (PtRu/C), using a convenient two-step solvothermal method. The constructed PtRu/C at different NaOH contents (0–1.25 mmol) were compared and electrochemical activity were evaluated by the hydrogen oxidation reaction (HOR). In contrast, the homogeneous distribution and minimum average size of Ru and Pt nanoparticles on carbon, appeared at approximately 4 nm, proving that PtRu/C-0.75 possessed abundant accessible active sites. The catalytic activities and the reaction mechanism were studied via electrochemical techniques. PtRu/C-0.75 has excellent activity due to its unique electronic structure and efficient charge transfer, with the largest j_0_ value of 3.68 mA cm^−2^ in the HOR.

## 1 Introduction

To protect the environment and solve the increasingly serious energy crisis, the development and application of green renewable energy have gradually become an emerging research field. However, the traditional energy industry is still dominated by fossil energy which causes many environmental problems such as air pollution. Therefore, environmentally friendly and energy-efficient fuels are desired. The fuel cell is currently one of the viable green energy technologies (Lu and Zhuang, 2016; Ohyama et al., 2016; Cong et al., 2018; Zhao et al., 2021). The anodic electrode catalysts used in fuel cells are still limited to Pt-based alloy electrode catalyst. Some catalysts that strive to substitute platinum carbon are faced with problems such as low catalytic activity, poor stability, and difficulty in large-scale preparation, which also have become numerous obstacles in the development of fuel cells. However, the current high cost of platinum carbon catalysts leads to little prospect of industrial application for fuel cells (Durst et al., 2014; Zheng et al., 2015). Therefore, the development of catalysts with good performance, strong stability, and low price are of great significance for promoting the promotion of fuel cells (Ishikawa et al., 2020; De Lile et al., 2021).

Among platinum group elements, Pt is currently the most studied platinum group metal (Gao et al., 2017; Weber et al., 2019; Pu et al., 2020). However, due to the scarcity and high cost of platinum, non-Pt metals nanoparticles have recently attracting increased attention. When the non-Pt metals are introduced, the amount of Pt can be decreased and the electronic state can be modified to optimize its electronic state, thus achieving high catalytic efficiency. There are a great number of works on Pt-based bimetallic electrocatalysts, such as alloying (Gao et al., 2017; Lee et al., 2017) Pt with 3d-transition metals, composing 3d-transition metals as core and Pt as shell (Gan et al., 2012; Gan et al., 2013). PtM (M = Fe, Co., Ni, etc.) bimetallic nanomaterials have been demonstrated to be promising electrocatalysts and for reducing Pt content. However, when such catalysts are exposed to the harsh operating conditions of fuel cells, complications arise. Platinum-group metals such as Au, Pd, Ir and Ru have much higher stability (Takimoto et al., 2017), but Ru is relatively less studied. Ru catalyst has the following advantages: high catalytic activity, stout oxidation ability of H_2_ in acidic medium, and strong stability (Bai, 2018); the price of Ru is cheaper than Pt, and Ru can further reduce the amount of Pt, thereby reducing the cost. Some studies have been carried out on Ru-based catalysts (He et al., 2019; Lao et al., 2021). Li and his co-workers have synthesized catalysts composed of Ru/C with higher mass activity and exchange current density than Pt/C (Li et al., 2019). Junya Ohyama et al. have prepared Ru/C catalyst with higher hydrogen oxidation performance by controlling the ruthenium particles at 3 nm (Ohyama et al., 2013).

Researchers attempted to add ruthenium atoms to Pt atoms to form alloys (Rodríguez-Gómez et al., 2022) or core-shell catalysts (Alayoglu et al., 2008; Hsieh et al., 2013; Elbert et al., 2015; Schwämmlein et al., 2018), mainly because ruthenium, has the strong electronic effect and oxygen-philic property, which can not only maximize the utilization efficiency of platinum but also enhance the activity of catalysts (Strmcnik et al., 2013; Wang et al., 2015; Zhang et al., 2021). During the catalyst preparation, Daisuke Takimoto et al. synthesized Ru nanosheets by surface limited redox replacement (SLRR) technique. Ru nanosheets served as the core for the synthesis of two-dimensional Ru-core@Pt-shell catalysts using the Cu underpotential deposition method (Cu-UPD) on mirror-polished glassy carbon rod (Takimoto et al., 2017). Xing et al. prepared Ru-rGO by liquid phase co-reduction method using NaBH4 as a reducing agent. Then Ru@Pt core-shell catalysts were synthesized on glassy carbon by UPD-GD approach (Xing et al., 2020). Cong et al. synthesized a series of uniform 3.0–3.8 nm Pt1−xRux particles supported on nitrogen-doped carbon (N-C) by wet-impregnation, high-temperature reduction, and high-temperature NH3 etching (Cong et al., 2020). However, the aforementioned catalysts have complex structure and preparation process, and the use of strong reducing agents such as NaBH4 will cause harm to human health. Moreover, the high cost and low surface-available active species, still restrict the industrial application of these catalysts.

Herein, nano-sized PtRu/C electrocatalyst with separated phases and high dispersion was produced using the method of twice hydrothermal. The resulting PtRu composite electrocatalyst was small in size and highly uniform in the carbon matrix, thus exposing more nanoparticles and providing abundant accessible active sites. The degree of dispersion and size of particles can be adjusted by changing the concentration of NaOH, which can produce PtRu/C with high intrinsic activity. With the presence of NaOH, the as-prepared PtRu/C provides very encouraging HOR performance, which can achieve 14.25 mA cm^−2^ at 100 mV overpotential in acidic. Because of these features, the resulting PtRu/C electrocatalysts showed excellent activities for the hydrogen oxidation reaction.

## 2 Materials and Chemicals

### 2.1 Experimental Section

Activated carbon (AC) was purchased from Sinopharm Chemical Reagent Co., Ltd. (China). Ruthenium chloride hydrate (RuCl_3_) and chloroplatinic acid hexahydrate (H_2_PtCl_6_.6H_2_O) were purchased from Macklin (Shanghai, China). Isopropyl Alcohol (C_3_H_8_O) (AR, 99.7%) and sodium hydroxide (NaOH) (AR, 96%) were purchased from Tansoole (China).

### 2.2 Preparation of Catalysts and Modified Electrodes

#### 2.2.1 Preparation of PtRu/C Catalysts

**FIGURE 1:**
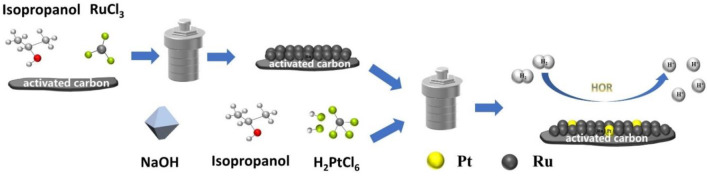
Schematic illustration for the synthesis of PtRu/C nano assemblies through the solvothermal process.

The preparation illustration of PtRu/C nano assemblies is shown in Figure 1. In the synthesis of Ru/C, activated carbon (0.2 g) was first dispersed in solution containing RuCl_3_ metal precursors (0.09 g) with isopropanol (16 ml) as the reductant and solvent. The solution was then transferred to a Teflon-lined autoclave with a stainless-steel shell and kept at 160°C over for 6 h. The black precipitates were washed with deionized water and ethanol and dried at 60°C for 12 h in a vacuum oven. Subsequently, the PtRu/C catalysts were prepared by solvothermal method again. H_2_PtCl_6_.6H_2_O(0.028 g), Ru/C (0.1 g) were dissolved in isopropanol (16 ml) and dispersed by ultrasonic agitation. To research the effect of different concentrations of NaOH on hydrogen oxidation, the NaOH concentration in the mixed solution was controlled at 0, 0.5, 0.75, 1, and 1.25 mmol. PtRu/C-0, PtRu/C-0.5, PtRu/C-0.75, PtRu/C-1 and PtRu/C-1.25 are respectively represented in the following sections. The solution was then transferred to a Teflon-lined autoclave with a stainless-steel shell and kept at 80°C over for 2 h. Finally, the resulting dark powder was sequentially washed with deionized water and ethanol successively, and then dried at 60°C for 12 h in a vacuum oven.

#### 2.2.2 Preparation of Rotating Disk Electrodes

Catalyst ink was prepared by mixing catalyst (4.0 mg), 5wt% Nafion (30 μl, DuPont Company), alcohol (300 μl), and deionized water (700 μl). The working electrode was a well dispersed catalyst inks (8 ul) rotated deposited onto glassy-carbon electrodes (A = 0.1256 cm^2^) during drying. The catalyst load on the modified electrode was estimated to be 0.0246 mg cm^−2^ (metal mass).

### 2.3 Physical Characterization

The structural characterization of the PtRu/C electrocatalyst were investigated using X-ray diffraction (XRD) and transmission electron microscopy techniques (TEM). Parameters were measured using an X'Pert3Powder (PANalytical, Netherlands) using Cu Kα (λ = 0.15, 406 nm) as the radiation source at 40 kV and 40 mA. By employing a JEM-2100F transmission electron microscope (resolution 0.19 nm), which has a high-resolution camera and an accelerating voltage of 120 kV, we were able to examine the catalysts. By using an X-ray photoelectron-spectrometer (ESCALAB 250Xi, United States) operating at 15 keV, we were able to determine the elemental composition of the catalysts used in this study. Based on the Brunauer-Emett-Teller (BET) and Barrett–Joyner–Halenda (BJH) equations, the specific surface area and pore size distributions of the nitrogen adsorption/desorption isotherms were analyzed using the Autosorb-iQ model.

### 2.4 Electrochemical Measurements

The HOR electrocatalytic testing was carried out using a standard three-electrode cell with a glassy carbon electrode as working electrode (S = 0.1256 cm^2^), saturated calomel (SCE) as the reference electrode, and a platinum slice as the counter electrode, respectively. Electrochemical measurements were performed with the model CHI660D electrochemical workstation. The CV was measured in H_2_-saturated H_2_SO_4_ at a scan rate of 50 mV s^−1^ when the gas was saturated with H_2_. Allowing the electrolyte to become saturated with pure hydrogen for at least 30 min, the polarization curves were recorded by sweeping the potential from −0.05 to 0.4 V at a scan rate of 10 mV s^−1^ while rotating the electrodes at varied rotation speeds (400, 900, 1,600, 2,500, and 3,600 rpm). At the open circuit potential, the electrochemical impedance spectroscopy (EIS) measurements were carried out at different frequencies, with frequencies ranging from 10^4^ to 1 Hz.

## 3 Results and Discussion

The preparation illustration of PtRu/C nano assemblies was shown in Figure 1. In the synthesis of Ru/C, we first dispersed activated carbon in the solution containing RuCl_3_ metal precursors with isopropanol as the reductant. Then, the solution was transferred to a Teflon-lined autoclave with a stainless-steel shell. The PtRu/C catalysts were prepared by the solvothermal method again. The acidity of a mixed solution of H_2_PtCl_6_.6H_2_O and isopropyl alcohol was controlled by the addition of NaOH to the solution, and Ru/C was added and dispersed by ultrasonic agitation. Finally, we obtained electrocatalyst as shown on the right side of Figure 1.

**FIGURE 2:**
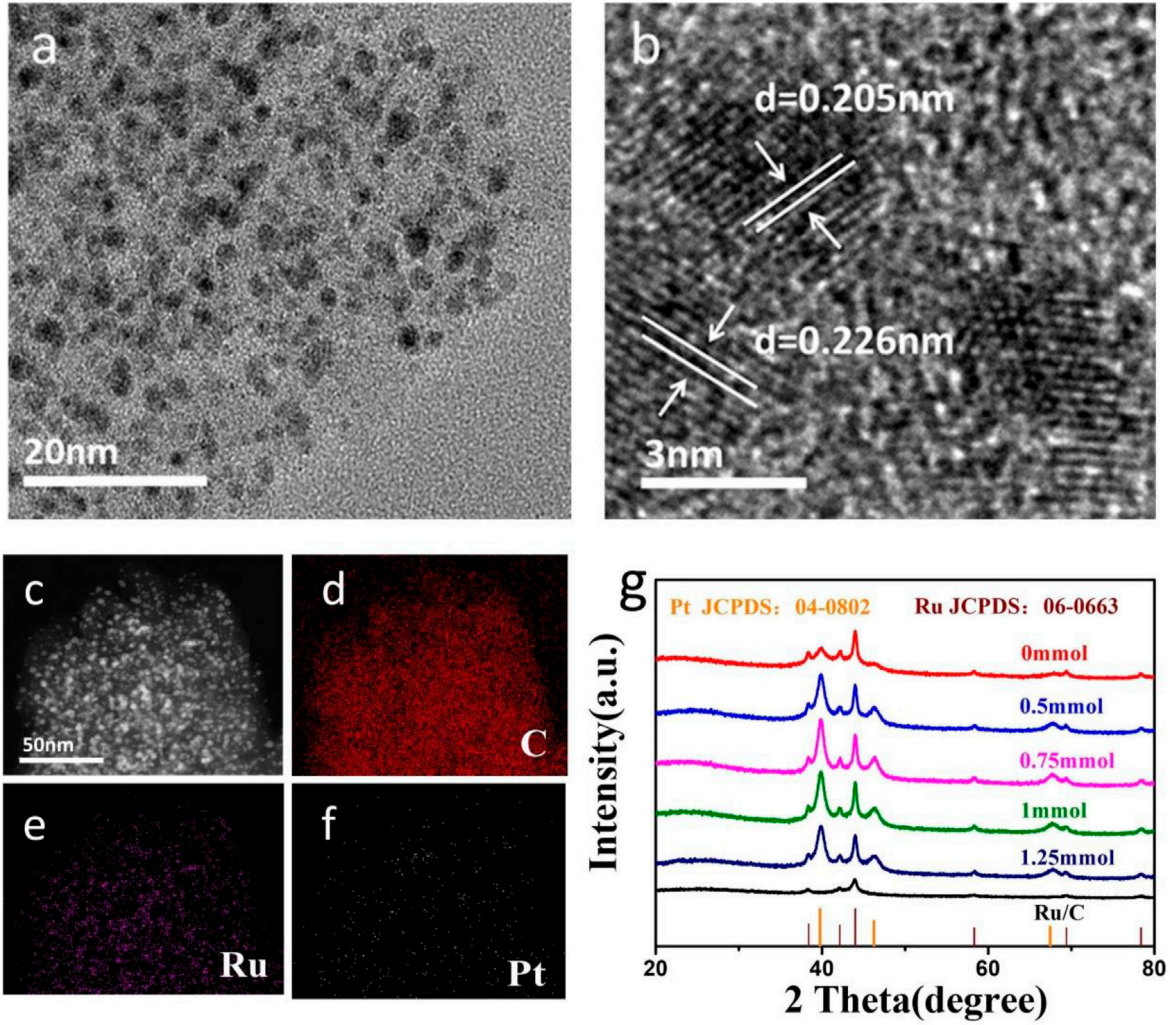
**(A)** TEM image of PtRu/C-0.75, **(B)** HRTEM image of PtRu/C-0.75, **(C)** STEM image of PtRu/C-0.75 and the corresponding EDX elemental mapping of **(D)** C, **(E)** Ru and **(F)** Pt, **(G)** X-ray diffraction (XRD) patterns of PtRu/C at different NaOH concentration.

The morphology and elemental composition of PtRu/C-0.75 were characterized by transmission electron microscopy (TEM). As shown in Figure 2A, the nanoparticles are homogeneously embedded in the amorphous activated carbon. The highly magnified images (HRTEM) shown in Figure 2B demonstrates the lattice fringe of PtRu/C-0.75. HRTEM images in Figure 2B reveal that the nanoparticles tend to form a separated phase since the determined d-spacing of 0.205 nm, which corresponds to the (101) planes of Ru, and other determined d-spacing of nanoparticles is 0.217 nm equal to Pt (111) (Lu et al., 2020). Due to the uniform distribution of Ru and Pt nanoparticles in the carbon, a unique phase is generated in the activated carbon, as shown in the mapping findings from the scanning transmission electron microscopic (STEM) diagram in Figure 2C.

The chemical composition and crystal structure of the PtRu/C were characterized using X-ray diffraction (XRD). The spectrograms of PtRu/C-0, PtRu/C-0.5, PtRu/C-0.75, PtRu/C-1, and PtRu/C-1.25 were illustrated in Figure 2G. The diffraction peaks of the samples appeared at 38.3°, 42.1°, 44.0°, 58.3°, 69.4°, and 78.4°, corresponding to the (100), (002), (101), (102), (110), and (103) planes of hexagonal structured Ru, respectively (Li, Abbott et al., 2019). The other peaks detected in the same sample were located at 39.9°, 46.4°, and 67.7°, respectively, belonging to the (111), (200), and (220) planes of face-centered cubic (fcc) structured Pt (Long, Wang et al., 2019). The XRD patterns showed obvious diffraction peaks of Pt and Ru, but the diffraction peak of C is not obvious, which may due to the amorphous form of carbon in the catalyst. It also can be confirmed that these catalysts are separated phases with multiple crystal structures. In the samples, the (111) main peak of Pt was more evident with the increase of NaOH content, which was mainly attributed to the exposed Pt nanoparticles. In contrast, PtRu/C-0 has insignificant Pt diffraction peaks. Remarkably, the 111) main peak of Pt gradually increased with the advance of the NaOH concentration and reached its peak with the concentration of 0.75 mmol.

**FIGURE 3:**
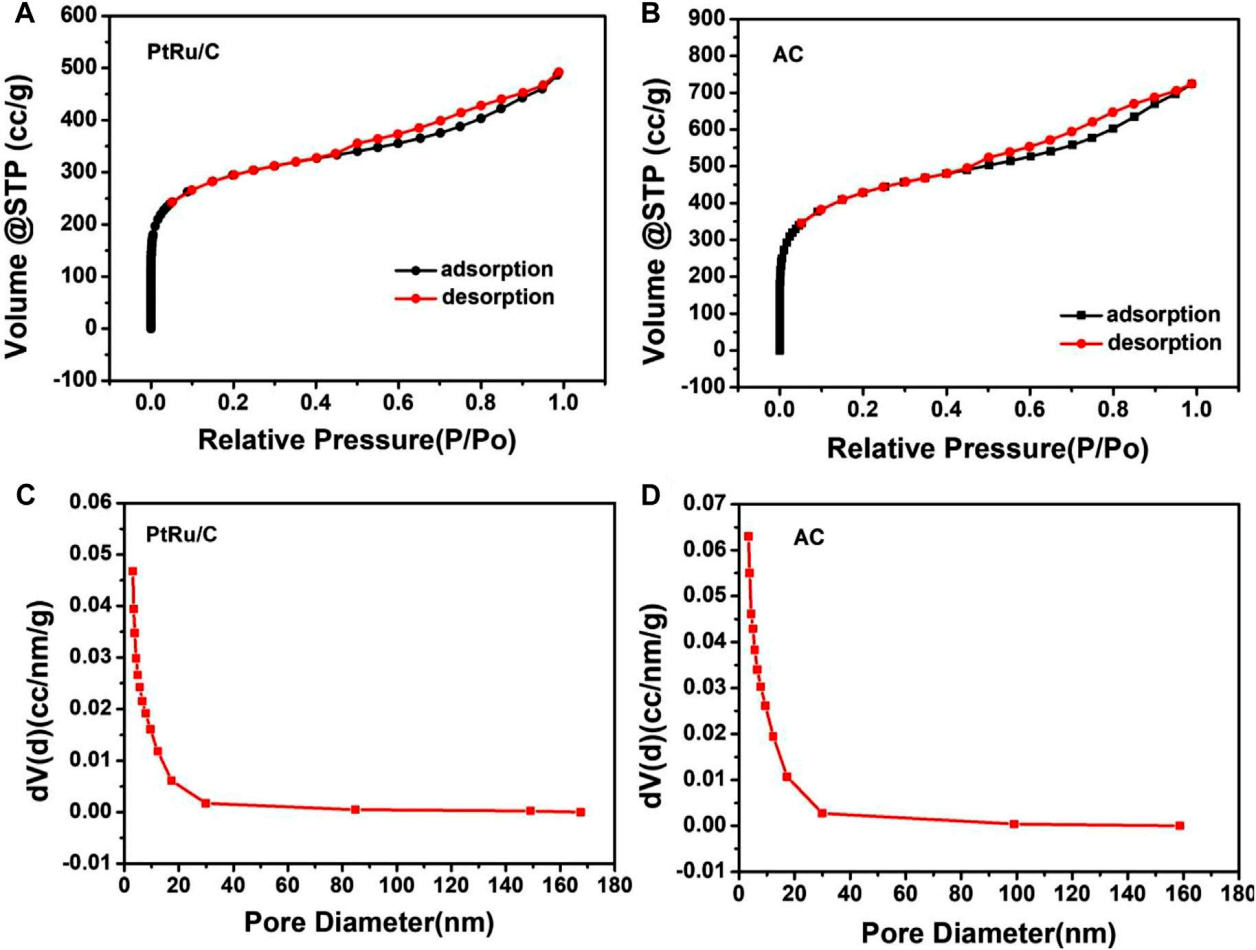
Nitrogen adsorption/desorption isotherms of **(A)** PtRu/C-0.75 and **(B)** AC samples, pore diameter distribution of **(C)** PtRu/C-0.75 and **(D)** AC samples.

As shown in Figure 3, the typical sorption isotherms of Type-IV with H4 hysteresis characterized the porous properties of both samples, with pore sizes ranging from micro to meso-range, which endow them with a high surface area of 167.53 and 234.74 m^2^ g^−1^, respectively (Khan, Sofian et al., 2020). Although part of the pores in AC were filled after loading the catalysts, resulting in a reduction in its specific surface area, the PtRu/C value was still higher than that of commercial ones (about 126 m^2^ g^−1^) (Jusys, Schmidt et al., 2002; Rodríguez-Gómez, Lepre et al., 2022). This loose structure is favorable for Pt and Ru to expose more active sites in PtRu/C, and enhances the catalytic performance for hydrogen oxidation reaction.

**FIGURE 4:**
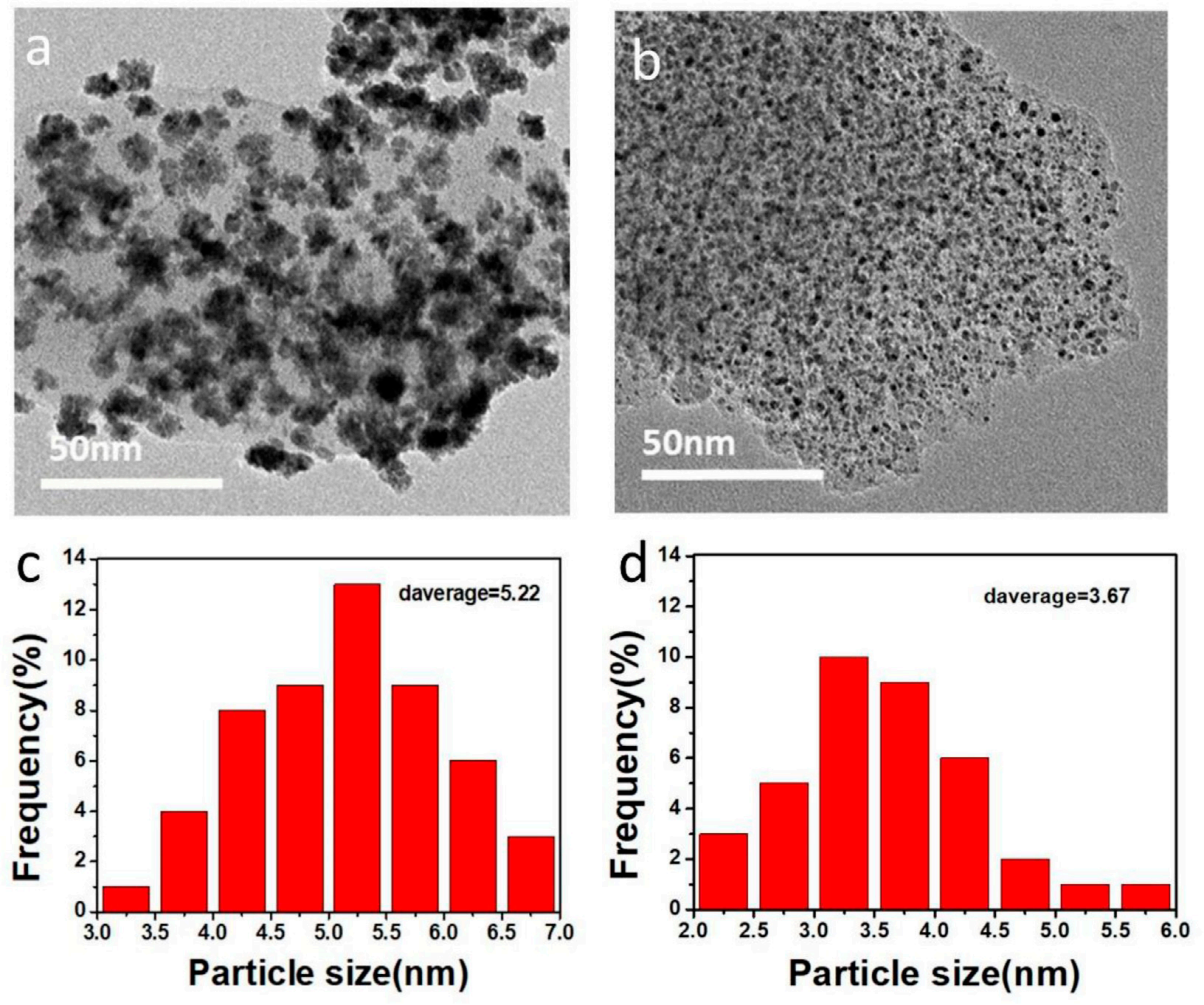
TEM images of **(A)** PtRu/C-0 and **(B)** PtRu/C-0.75 samples, corresponding size distribution histograms of **(C)** PtRu/C-0 and **(D)** PtRu/C-0.75 samples.

The transmission electron microscope images and particle distribution histograms of PtRu/C catalysts synthesized under different NaOH concentration conditions are shown in Figure 4. It can be demonstrated that the PtRu/C catalyst particles prepared with a certain concentration of NaOH are approximately spherical and evenly distributed on the carbon carrier without obvious agglomeration in Figure 4B. In contrast, as shown in Figure 4A, the PtRu/C catalyst particles prepared without NaOH are seriously agglomerated and unevenly distributed. The particle size distribution histograms were drawn after measuring the particle size of 200 particles in a random area of TEM images. The average particle size of metal particles in PtRu/C-0.75 catalyst was 3.677 nm, which was smaller than that of PtRu/C-0 catalyst (5.22 nm), as shown in Figures 4C,D. With the decrease of the size of nanoparticles, the atomic coordination number on the surface of catalyst particles decreases, on the contrary, the number of active sites increases. Overall, the particle size of PtRu alloys decreases as the atomic percent of Ru increases, since NaOH plays a critical role in controlling the size of the PtRu nanoparticles rather than affecting their crystal structure (Xu et al., 2021).

**FIGURE 5:**
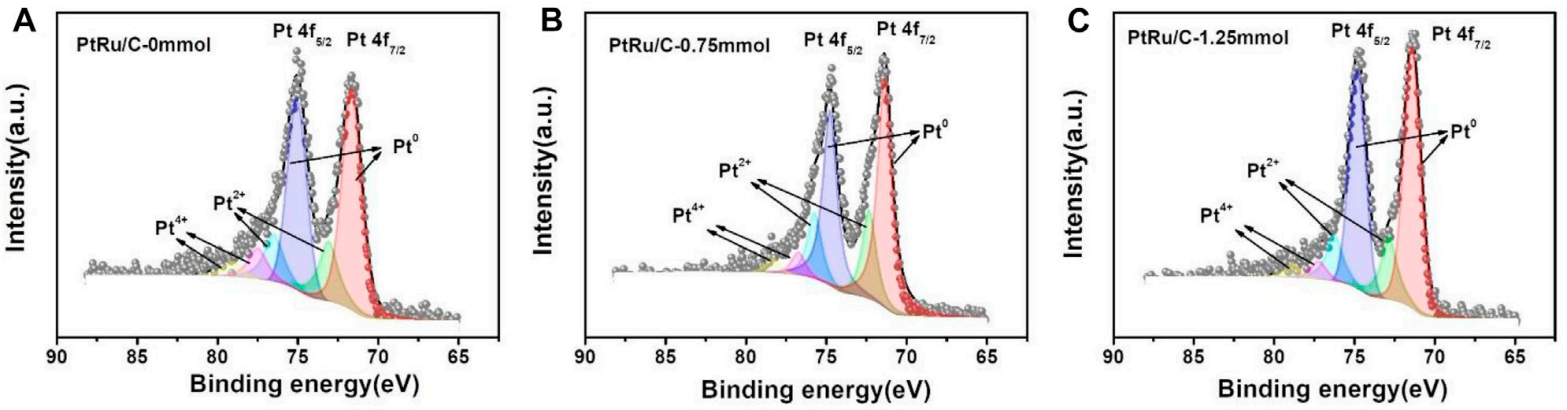
XPS spectra of Pt 4f of **(A)** PtRu/C-0 and **(B)** PtRu/C-0.75 **(C)** PtRu/C-1.25 samples.

The surface species and chemical states of PtRu/C nanocomposites were characterized by X-ray photoelectron spectroscopy (XPS), as shown in Figure 5. The characteristic peaks of Pt, Ru, and C were observed in the PtRu/C-0, PtRu/C-0.75, and PtRu/C-1.25 samples, indicating the successful fabrication of bimetallic samples. The high-resolution spectra of Pt 4f for the three PtRu/C samples, which could be split into two pairs of doublets, are shown in Figures 5A–C, respectively. The lower binding energy peak in each sample correlates to the Pt 4f_7/2_ orbit, whereas the other corresponds to the Pt 4f_5/2_ orbit. In previous research, it was discovered that the typical energy of the Pt 4f_5/2_ (Table 1) peak of a pure Pt samples was 71.2 eV (Zhang et al., 2021). The table is the binding energy of the peaks of different valence states of the Pt element of the samples. Furthermore, in PtRu/C-0, PtRu/C-0.75 and PtRu/C-1.25 samples, the peaks of Pt^0^ have slightly higher BEs by 0.44, 0.16 and 0.2 eV for 4f_7/2_, respectively, than the typical energy of the Pt 4f_5/2_ peak of pure Pt samples. The two sets of peaks with higher BEs are assigned as the 4f_7/2_ and 4f_5/2_ peaks of Pt^2+^ [PtO or Pt (OH)_2_] and Pt^4+^ (PtO_2_), respectively (Matin, Lee et al., 2015). As expected, the binding energy of the three samples produces a positive shift relative to the Pt itself, which indicates the downward shift of the Pt d-band center, and the electrons of Pt 5d transfer to 4d orbital is not filled by Ru, and the holes of the 5d orbital of Pt increase, so the binding energy of the orbital electrons increases. Among the three samples, the ∆BE of PtRu/C-0.75 is the smallest, this suggests that PtRu/C-0.75 more prone to separate phases. Due to the fact that metallic Pt is the most important active site in the H-H cleavage, PtRu/C electrocatalysts block hydrogen adsorption and enhance H-H cleavage, and therefore can be expected to have better electrocatalytic kinetics than Pt alone. XPS analysis further confirmed the strong electron transfer interaction between Pt atom and Ru atoms, which indicates that Ru might affect the electronic structure of Pt, and the smaller size of Ru atom compress the lattice structure of Pt. From the table, we can see the peak area ratios of different valence states of Pt for samples. The peak area of Pt^0^ of the three samples has a relatively large proportion, and the ratio of the Pt^0^ peak area of PtRu/C-0.75 and PtRu/C-1.25 is slightly more than PtRu/C-0. It shows that adding NaOH can better reduce the precursor platinum chloride.

**TABLE 1:** The binding energy of the peaks of different valence states of the Pt element of the samples.

Sample BE	PtRu/C-0	PtRu/C-0.75	PtRu/C-1.25
Pt^0^ 4f_7/2_	71.64 eV	71.36 eV	71.4 eV
Pt^0^ 4f_5/2_	75.04 eV	74.75 eV	74.8 eV
Pt^2+^ 4f_7/2_	73.07 eV	72.32 eV	72.82 eV
Pt^2+^ 4f_5/2_	76.48 eV	75.72 eV	76.22 eV
Pt^4+^ 4f_7/2_	77.48 eV	76.74 eV	77.20 eV
Pt^4+^ 4f_5/2_	79.07 eV	78.32 eV	78.82 eV
∆BE (Pt^0^ 4f_7/2_)	0.44	0.16	0.2
Peak area ratio (0:2+:4+)	2.79:1:0.42	2.57:1:0.26	3.11:1:0.3

**FIGURE 6:**
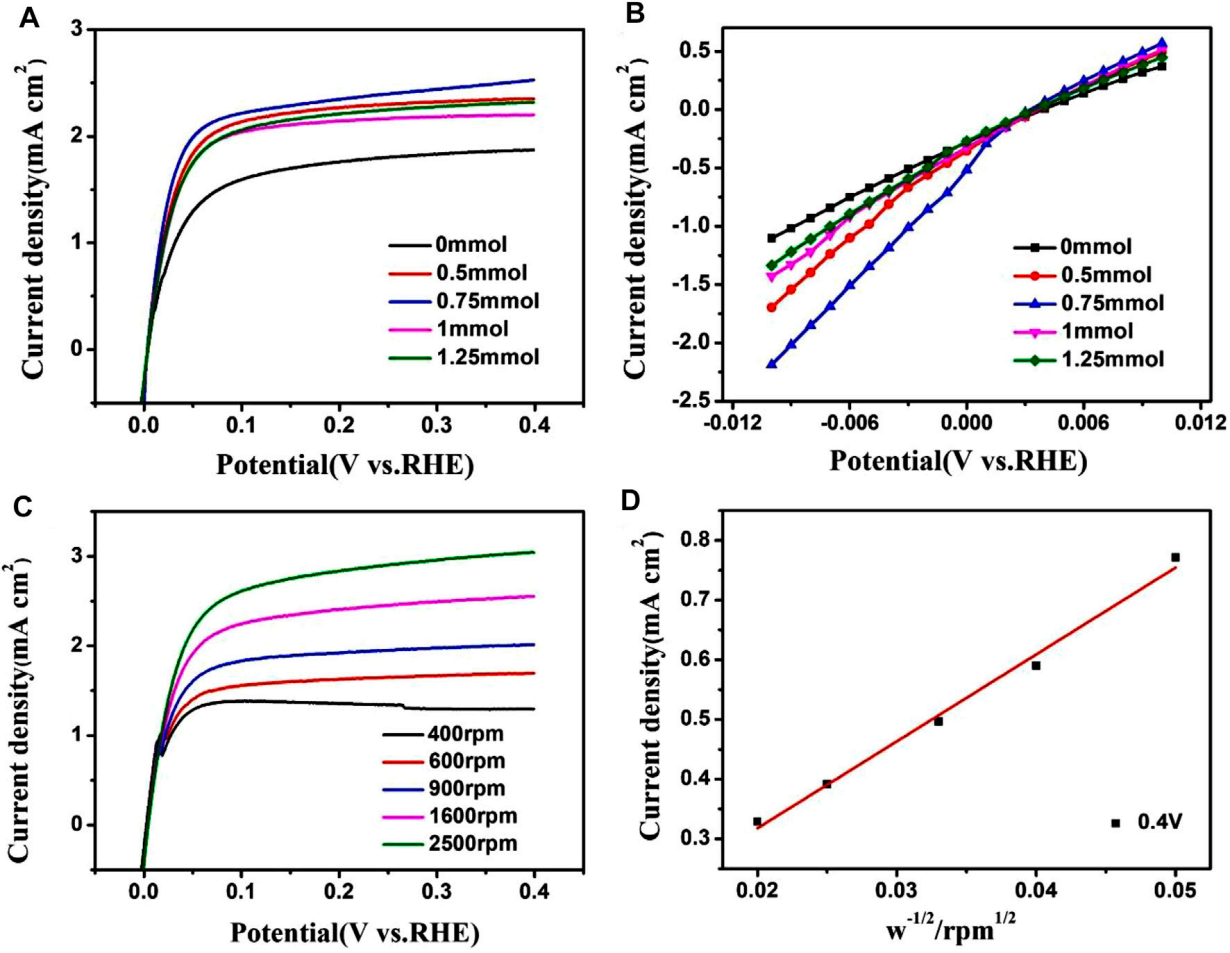
**(A)** Polarization curves of PtRu/C catalysts in 0.1 M H_2_SO_4_ aqueous solution saturated with H_2_ at a scan rate of 10 mV s^−1^and rotating speed of 1,600 rpm. **(B)** The linear-current potential region around the equilibrium potential for hydrogen oxidation/reduction. **(C)** Polarization curves of PtRu/C-0.75 catalysts in 0.1 M H_2_SO_4_ aqueous solution saturated with H_2_ at a scan rate of 10 mV s^−1^ with various rotating speeds. **(D)** Kentucky-Levich plot at an overpotential of 0.4 V.

The hydrogen oxidation reaction performance was evaluated with a rotating disk electrode, and all currents were normalized to the geometric surface area of the electrode. Figure 6A shows the polarization curves of PtRu/C catalysts in 0.1 M H_2_SO_4_ aqueous solution saturated with H2 at a scanning rate of 10 mV s^−1^ and a rotating speed of 1,600 rpm. The ECSA normalized exchange current densities (j_0_) were calculated to compare its catalytic activity. The exchange current was evaluated in the micropolarized region with deviations of only a few millivolts from −0.01 to 0.01 V.

$$j=j_{0}\left(\eta F/RT\right)$$

where η is the applied overpotential ( |η| ≤ 0.02 V), R is the gas constant [8.314 J mol^−1^ K^−1^)], and F is the Faraday constant (96,485 C mol^−1^).

Moreover, we can get the exchange current densities: in PtRu/C samples, 3.68 mA cm^2^ (0.75 mmol) > 2.79 mA cm^2^ (0.5 mmol) > 2.48 mA cm^2^ (1 mmol) > 2.3 mA cm^2^ (1.25 mmol) > 5.28 mA cm^2^ (0 mmol). Of the PtRu/C catalysts studied, the PtRu/C-0.75 sample showed the largest exchange current density (3.68 mA cm^−2^). The PtRu/C catalyst has a high current density, which thus increases the electrochemical activity of hydrogen oxidation reaction (HOR). There is a clear correlative dependence between activity and chemical composition, with the PtRu/C-0.75 catalyst demonstrating the highest exchange current density of 3.68 mA cm^−2^. When NaOH is added during preparation, metal ions often coordinate with OH^−^ to form mixed M-OH intermediates with reduced redox potentials, thereby reducing the redox reactions rate, and forming small-sized NPs(Xu, Chen et al., 2021). TEM and BET results showed that the small metal particles had a relatively large specific surface area, thereby increasing their hydrogen oxidizing activity. The Levich plot shown in Figure 6D exhibits a linear relationship between the inverse of j_0_ and ω^1/2^. The calculated slope is 0.068 mA cm^−2^_disk_rpm^0.5^, which is very close to the theoretical value (Mao et al., 2020). The hydrogen oxidation current density of PtRu/C is comparable to the activity of catalyst reported in the previous literature (Table 2). The addition of Ru promotes a more effective and prominent electronic effect, as shown by as suggested by the XPS results, is a particularly important and influential variable in determining HOR activity.

**TABLE 2:** Comparison of the performance of HOR in acid media.

year	Electrocatalyst	Loading (µg cm^−2^_disk_)	Exchange Current density (mA cm^−2^)	Ref
2016	Pd-CN_X_	43	0.84	Bhowmik et al. (2016)
2016	Pt (commercial)	51	1.14 ± 0.04	Hunt et al. (2016)
2016	PtRu (commercial)	51	1.14 ± 0.04	Hunt et al. (2016)
2016	PtRuc-s	20	1.11 ± 0.03	Hunt et al. (2016)
2010	Pd/C	7.5–15	0.25	Pronkin et al. (2010)
2022	Pt/TiO_2_(25 %R)-CNx	8	0.962	Cui et al. (2022)
2021	PtRu/C	24	3.68	This work

**FIGURE 7:**
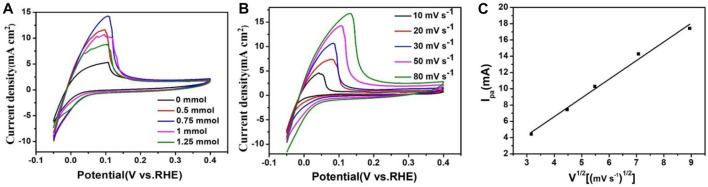
**(A)** CVs of PtRu/C catalysts in 0.1 M H_2_SO_4_ aqueous solution saturated with H_2_ at a scan rate of 50 mV s^−1^ **(B)** CVs of PtRu/C catalysts in 0.1 M H_2_SO_4_ aqueous solution saturated with H_2_ at various scan rates. **(C)** Linear graph of scanning speed and peak current.

The CV of PtRu/C in 0.1 M H_2_SO_4_ (Figure 7A) features a redox peak centered at ∼0.05 V in the anodic scan followed by a tapering shoulder up to ∼0.1 V, which was caused by the H_upd_ desorption of the catalysts as well as the oxidation of Ru species (Ramaswamy et al., 2017; Ishikawa et al., 2020). In the previous studies, the potentials of the H_upd_ peaks correspond to the HBE values under the test conditions (Lu and Zhuang 2017). The emergence of H_upd_ peaks for the PtRu/C-0.75 at lower potential indicates the existence of weaker hydrogen bonding sites. The weakened hydrogen binding energies for the samples are due to the electronic and geometry effect from the Ru and Pt additive. We can obtain the hydrogen oxidation current density in PtRu/C samples, 14.28 mA cm^2^ (0.75 mmol) > 11.42 mA cm^2^ (0.5 mmol) > 10.43 mA cm^2^ (1 mmol) > 8.57 mA cm^2^ (1.25 mmol) > 5.28 mA cm^2^ (0 mmol). It is worth noting that the current density of the catalyst gradually increased with the increase of NaOH concentration, and reached the peak at the concentration of 0.75 mmol. According to the results of TEM, particle size, and XPS, we can conclude the reason why the hydrogen oxidation reaction (HOR) is the best at 0.75 mmol. The average size of PtRu/C samples (0.75 mmol) is the smallest (3.67 nm) and has a high dispersion, resulting in the electrocatalyst has a rich exposure site and the nanoparticle and “electronic effect”. PtRu/C samples (0.75 mmol) have the smallest average size (3.67 nm) and high dispersion, which leads to rich exposure sites, nanoparticles and “electronic effects” of electrocatalysts. Figure 7B shows the CV of PtRu/C-0.75 catalysts saturated with H_2_ at different scanning rates in 0.1 M H_2_SO_4_ aqueous solution. The current density of the PtRu/C-0.75 increased the scanning rate increased from 10 mV s^−1^–80 mV s^−1^. Figure 7C shows that the square root of the catalyst scanning speed was proportional to the peak current, indicating that hydrogen was adsorbed on the surface of the catalyst, and Ru and Pt as active centers on the surface of the catalyst which had a strong interaction with hydrogen.

**FIGURE 8:**
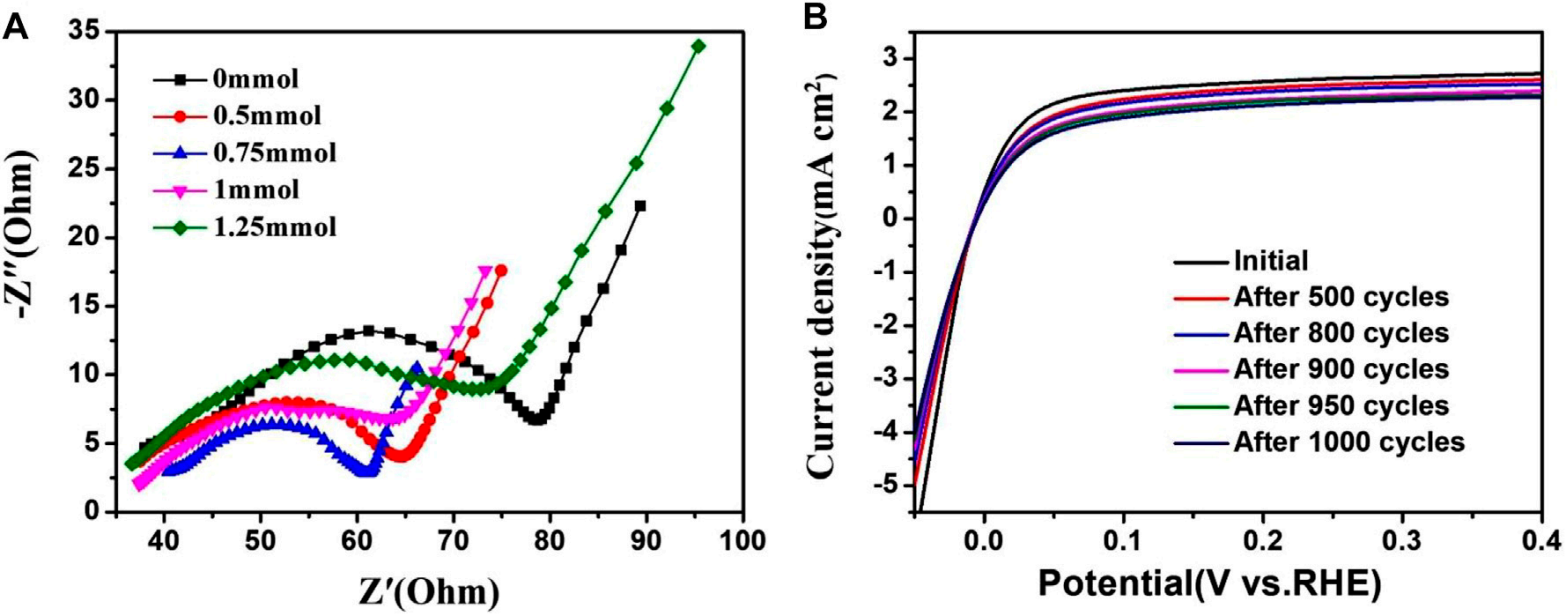
**(A)** Nyquist plots of PtRu/C catalysts **(B)** Polarization curves of PtRu/C-0.75 before and after 1000 CV cycles.

Electrochemical impedance spectroscopy (EIS) was carried out, from 10 kHz to 1 Hz, to gain a better understanding of the HOR kinetics occurring at the electrode/electrolyte interface. The Nyquist plots (Figure 8A) were fitted using an equivalent circuit to calculate the charge transfer resistance (Rct) of 51.37, 34.36, 23.42, 38.63 and 46.28 Ω for PtRu/C-0, PtRu/C-0.5, PtRu- 0.75, PtRu/C-1, PtRu/C-1.25. This indicates an efficient charge transfer at the interfaces and improved HOR kinetics for the PtRu/C-0.75 catalyst. The smallest Rct may be attributed to the separated compound that can facilitate the electron transfer process. As shown in Figure 8B, negligible change was observed in the HOR activity of PtRu-0.75 before and after 1000 CV cycles, indicating the excellent durability of PtRu-0.75.

## Conclusion

In this study, PtRu/C was effectively synthesized, with an average particle size of around 4 nm. According to the results of physicochemical characterization and electrochemical test, the catalyst exhibited the highest electrochemical activity on the hydrogen oxidation reaction when the concentration of NaOH reached 0.75 mmol, which was attributed to the small particle size, uniform dispersion, and electronic effect. Meanwhile, as compared to prior studies, the activity of hydrogen oxidation increased as a result of the development of catalyst. As a result, the catalyst is easy to produce and is a good option for proton exchange membrane fuel cells in the future.

## Data Availability

The datasets presented in this study can be found in online repositories. The names of the repository/repositories and accession number(s) can be found in the article/Supplementary Material.
